# Gynecological and Obstetrical Society of Baltimore

**Published:** 1891-05

**Authors:** William S. Gardner

**Affiliations:** Secretary; 410 Hanover St.


### GYNECOLOGICAL AND OBSTETRICAL SOCIETY OF BALTIMORE.

February meeting.

The President, Dr. Henry M. Wilson, in the chair.

Dr. Neale reported the following case of

### OCCLUSION OF THE OS UTERI DURING FOUR DAYS' PARTURITION.

Mrs. K. W., aet. 26 years, white—1 para. Past history unimportant. Last menstruation, early part of April, 1890. Pregnancy normal up to November 16, 1890, when she slipped and fell violently on her right side on the sidewalk. There was no vaginal discharge at the time and no discomfort except from the jar, bruisings, etc., and the patient was up and about all the time. The movements of the child were felt after the fall.

About Christmas, 1890, an offensive yellowish vaginal uterine discharge occurred and continued for one week.

On the night of January 12th, 1891, her first labor pains began and were so severe as to require morphine given by her attendant. There was no "show" or discharge of any kind. The pains increased and the patient was suffering severely when I saw her for the first time, Friday evening, January 16th, 1891. She was a large, well-built and well nourished woman.

Could not distinctly map out the child by abdominal palpation. By auscultation gurgling over the entire uterine tumor, and not a trace of fœtal heat sounds could be heard. By vaginal examination: very short and small vagina, no cervix and no os! A continuous layer of mucous membrane, flush with the vaginal walls, closed over the entire vault of the vagina and a little dimple in its centre was the only indication of where the os ought to be.

Patient chloroformed, placed in position, hand passed into vagina, finger pressed firmly against the dimple, when it suddenly yielded or burst open like a membranous web—permitting a gush of *not* foul smelling, bloody water to escape, and at once the rapidly enlarging outlines of the os could be felt, then about as wide as a silver half-dollar piece. The soft bagging scalp and loose cranial bones came down upon the enlarging os, and as the expulsive efforts were almost *nil*, I grasped the head with a Simpson's cranioclast, which tore away, and then the blades of a Tornier basiotribe were adjusted over the head and neck, and a thoroughly macerated but not decomposed or foul small child was easily extracted. Perineum intact—os fissured slightly. Small placenta expressed within six minutes. Considerable *post partum* hemorrhage, uterus acting feebly. Os remained open about size of silver half dollar piece, thick edges, uterus rather small but not firmly retracted. Two quarts of a hot intra-uterine 1-4000 bichloride douche were injected. Patient rallied well, and debarring an occasional slight rise of pulse and temperature, and faintly fœtid lochia, which readily yielded to the antiseptic douche, the puerperium was uneventful and recovery complete. This case was a novel one to me. I am quite sure the membrane I felt was mucus, and not the amnistic sac, nor do I think the case should be classed among those of cervical occlusion or stenosis from endotrochelitis.

DR. J. WHITRIDGE WILLIAMS read a paper on "The Induction of Premature Labor in Contracted Pelves." He pointed out that the comparative neglect of the operation in this country was due to two causes: the absence of large lying-in institutions and the consequent lack of large amounts of clinical material, and the almost total neglect of pelvic measurement.

By the term premature induction of labor one understands the artificial interruption of pregnancy at such a period that a viable child may be born; that is any period from the 28-30th week to the end of pregnancy.

Dr. W. then went into the history of the operation and showed that it was first rationally employed for this indication in England, as the result of a conference of the eminent physicians of London in the year 1756.

Within fifty years it was quite generally employed on the continent, and soon enjoyed a popularity which caused it to be resorted to on the most trifling pretexts, and which in 1869 called forth Spiegelberg's forcible denunciation of the operation by which he showed that the mortality both of the mothers and children was nearly three times greater after the operation than if the woman went on to term. This was followed by articles by Litzmann and Dohrn, who showed that Spiegelberg had painted the picture in colors far too dark.

Litzmann showed that in moderate degrees of contraction, 8.25—7.5 cm. (3¼–3in.), the operation was indicated in the interests of the mother, as shown by a mortality of 7.4 per cent. after the operation, compared with one of 18.7 per cent. when the woman was allowed to go on to term.

Dohrn stated that the proper method of appreciating what the operation accomplished was not to compare as many cases of induced labor with so many cases of labor at term, but to compare the results of premature and spontaneous labors in the same woman; by this method he found that twice as many children were saved by inducing labor as by allowing the woman to go on to term.

Consequently they proved that the operation was indicated in properly selected cases, both in the interest of the mother and child.

The introduction of antiseptic methods into midwifery almost completely robbed the operation of danger for the mother, as will be readily seen from the following statistics: Thus, Haidlen reports forty-four cases from the Stuttgart clinic, with no maternal deaths, and 72 per cent. of the children saved.

In 1889, Korn stated that Leopold lost one woman in forty-five cases and saved 66 per cent. of the children; and last July Ahlfeld stated that he had induced labor 118 times with the loss of only one mother, and had saved 62 per cent. of the children.

At the Berlin Congress, Fehling stated that in 60 cases he had saved all the mothers and 80 per cent. of the children.

From the above sketch we will readily see that the maternal mortality in properly selected cases is very light, four hundred and one cases collected by Korn showing a maternal mortality of only 2.9 per cent., or just a trifle more than a normal labor in a normal pelvis, while the fœtal mortality ranges from 20 to 70 per cent., the average being about $33\frac{1}{3}$ per cent. So in this operation we have a means of saving about two-thirds of the children without any risk to the mother. Or reckoning by Dohrn's method we save at least twice as many children as if we allowed the woman to go on to term, and then resorted to some conservative operation. These are the prospects of the operation; unfortunately the degree of contraction within which the operation is justifiable is very limited, and we can only think of it in moderate degrees of contraction. According to Litzmann, in flattened pelvis, with a conjugata vera of 7.5–8.25 cm.; (3–3.25 in.); and to Schrœder, 6.5–9.5 cm. (2.5–3.75 in.).

As pelves with a conjugata vera above $8\frac{1}{2}$ cm. ($3\frac{3}{8}$ in.) offer a reasonable chance to both child and mother at term, and those below 7 cm. ($2\frac{3}{4}$ in.) offer no chance to the child, I think that the operation should be restricted to these limits; that is, between 7–$8\frac{1}{2}$ cm. ($2\frac{3}{4}$–$3\frac{3}{8}$ in.), in simple flattened pelves.

In the justo-minor pelvis, a conjugata of $9\frac{1}{2}$ cm. ($3\frac{3}{4}$ in.) or less will usually be an indication for the operation. In the rare forms of obliquely narrowed pelvis, whatever its cause, we must be guided almost entirely by the history of previous labors.

We thus have the operation restricted to a very small range—$1\frac{1}{2}$ cm. ($\frac{5}{8}$ in.), which should only be exceeded when the previous history tells us that the previous labors have all ended disastrously. We should not think of inducing labor in a flattened pelvis with a conjugata below 7 cm. ($2\frac{3}{4}$ in.), for in that case the prospects for the child are almost *nil*, and the danger to the mother greatly increased. Here we come to the relative indication for Cæsarean section, when it is best to allow the woman to go on to term, and attempt to save the mother and child by that operation.

With these contracted indications, we readily see that an accurate idea as to the exact size and form of the pelvis is an absolute prerequisite for the performance of the operation; and the only means by which we can accurately obtain this information is by carefully measuring the pelvis.

We should not content ourselves with simply measuring the conjugata vera, but should also take the external measurements and thereby attempt to determine with what form of pelvis we have to deal. After doing that, we must carefully examine the interior of the pelvis to determine its height; to see if it is generally contracted, and if contracted, if the contraction increases as we approach the outlet, we must look for exostoses of the pelvic bones and carefully examine the promontory to see if it is double or not.

If we think the pelvis contracted laterally we should measure the distance between the tubera ischiorum on each side, as Breisky recommended. We should also attempt to estimate the transverse diameter of the pelvis, which is most difficult to do, and the most that can be expected is to examine alternately with each hand and try to stroke the linea innominate, and so relatively get some idea as to the transverse diameter.

Having decided that an operation is necessary the next question is, when shall it be done? Of course, the younger the fœtus the smaller will be its size, and consequently the easier its delivery. But unfortunately, the smaller the fœtus, the less chance will it have of living even if it survive the operation. Generally speaking, we say a child is viable after the 28th week, but its chances of living are almost *nil*; indeed children thirty to thirty-two weeks old have next to no chance of living. The earlier the operation, the more chance has the fœtus of living after it, but unfortunately its size and consequently the difficulty of its delivery increase with its age. If possible, the operation should be done about the 34th to 36th week, our object being to operate at the latest possible period consistent with safe delivery.

To fulfill this object we must attempt to gain accurate knowledge as to the size of the child's head. Unfortunately we are unable to determine its size with mathematical precision, or even with the relative precision of pelvimetry; so we are obliged to take advantage of every possible hint on the subject. Some of the following points may be of assistance in different cases. We must consider the mother's account as to the duration of pregnancy. Notice the size of the parents, large parents usually having large children. Inquire about the previous labors, particularly as to the size of the head. Endeavor to estimate the size of the head by abdominal and combined abdominal and vaginal palpation; and note the consistency and amount of resistance to compression that the bones of the head offer.

Try to measure the head with the pelvimeter through the abdominal walls, and deduct the estimated thickness of the abdominal walls from the result.

Notice the size of the large anterior fontanelle, average width 2 cm.; the width of the sutures, and the distance from the anterior to the posterior fontanelle; for as they are larger or smaller, it indicates a larger or smaller head. Measure the length of the fœtus as it lies *in utero*, from breech to vertex, double the measurement and it gives, according to Ahlfeld, the length of the fœtus. If a foot is prolapsed, measure it, for Goenver stated that there is a difference of nearly 1 centimeter between the length of the foot of a child at term and one at thirty-two to thirty-four weeks.

One of the most important methods is that of Mueller, who attempts to force the head down into the pelvis by pressure from above. As long as he is able to force the head down, he knows that labor will readily take place; but when he can no longer force the head down and when it bulges out over the symphysis, then he considers that the time for operation has arrived. As the great danger to the mother is from sepsis, one cannot be twó careful in one's efforts to guard against it, and consequently one should be most particular in ones's preperation for the operation.

For several days previous to operating, the woman should have a warm bath daily; and several times a day be douched with warm water, 95°- 98° F. containing salt or borax by which the cervix is softened and dilated. Just before operating, the genitals should be most carefully washed with hot water and soap, followed by a 1–1000 bichloride solution; the vagina should also be most carefully cleaned.

The hands of the operator should be washed for at least ten minutes in hot water and the nail-brush vigorously used, after which they should be placed for several minutes in a 1–5000 bichloride solution.

All instruments should be sterilized by strain or placed in a five per cent. solution of carbolic acid for at least thirty minutes.

The most generally approved method is that of Krause or the introduction of a disinfected flexible bougie between the membranes and the uterine wall. If properly conducted it is almost entirely devoid of danger for the mother, and will bring abo . the birth of the child in a period varying from 8–214 hours, averaging about 80 hours or about three days. To insert the bougie, the woman is placed on her back or side as may be most convenient, and the cervix brought down by a pair of bullet-forceps and the cervical canal carefully cleansed with bichloride on a pledget of cotton; the bougie is then carefully inserted so that its lower end is within the vagina, care being taken not to wound the membranes or the placenta. Then the vagina is packed with iodoform gauze, care being taken not to wound the cervix which serves to hold the bougie in place. If at the end of twenty-four hours no labor pains have been produced, the bougie should be removed and another introduced at another point under the same precautions as the first.

If this method fail, we may resort to Kisvisch's method of allowing a current of hot water, 100°–110° F., to flow through the vagina several times a day for a period of 5–15 minutes, or we may puncture the membranes; as accessory to these, we may loosen the membranes about their lower pole, dampen the vagina with iodoform gauze or employ Barne's bags.

If the pains are weak, Fehling recommends version by Hick's method and bringing down one leg, whereby increased contraction is produced, and one is afforded a ready means of ending the labor if one deems it expedient in the interests of the mother or child.

DR. NEALE: I regard the chief point in this very able paper to be the endeavor to definitely fix the limits for the induction of premature labor in contracted pelves, not as opposed to Cæsarean section, but as applicable to a distinct and separate class of cases. This endeavor I strongly advocate, but at the same time must confess that I do not believe the plan is always practicable at the bedside. There are so many factors entering into the determination of this question, as I stated in my paper, that I can now only repeat what I there quoted, viz.: "A given pelvic measurement is useful as an indication of what has been the experience of others under similar circumstances, but is not a final ground for decision."

After this evidence adduced, which doubtless represents the opinion of the best medical authorities, I am sure I only voice the concurrence of this society in accepting the limits for this operation as stated by Dr. Williams.

This is practically in accordance with the teachings of Lusk, probably our strongest American authority, who places the range for the induction of premature labor in contracted pelves at a conjugata vera of from 2¾ inches (7 cm.) to 3½ inches (8.75 cm.)

As stated in the paper, I believe the most reliable statistics of this operation are those of Dohrn, who compares the results of induction of premature labor with those of labor of term in the same case, showing a very decided advantage in premature labor. It must be remembered, however, as Litzman has clearly shown, that children born alive by this operation are far more likely to die early than matured children. The risk to the child does not cease with the delivery.

I cannot recall any reference in the paper to pelves contracted from hip disease, and yet I have met with two obstetrical cases of this character during the past two years in this city; both were in private practice, and both were primiparæ.

The first case I saw in consultation during a very severe labor at term, and delivered her of a still born child by a difficult high (Tarnier) forceps operation.

Premature labor was induced on the second case at the eighth month. In this case the bougie was retained under antiseptic precautions (2 p. c. creoline cervical and vaginal douche and iodoform gauze over os) between the membranes and uterine walls for 48 hours without effect. It was then withdrawn, the douche again administered, and bougie reintroduced in a different position and retained for 24 hours again without effect. The sac was then punctured high up by the probe, and labor began in about 15 hours. Thus we see the method of Krause, although the best, may fail, where puncture of the sac will not.

As this lady was poisoned to death by an unclean servant who dressed and picked carious bone from her foot and then attended my patient, and handled all her linen, napkins, etc., without my knowledge, it shows the importance of extending our antiseptic precautious to everything coming in personal contract with the case.

As regards the method of delivery, the experiments of Budin and others speak strongly in favor of version and extraction as opposed to forceps.

DR. KELLY: The subject is too large to be discussed formally. I will merely refer to one or two points of interest. A serious complaint is to be entered against the records of foreigners in regard to the statistics of infant mortality after premature labor. Many observers only state whether the child was born living or dead; some few state whether or not it was living when discharged from the hospital. What we want to know for practical purposes, is whether the children live any time after they get home. My own experience is but few live. If they are sent out simply to die soon after at home, the induction of premature labor among the poorer classes simply becomes a species of uterine gymnastics.

A method of my own, which I have found most successful in inducing premature labor, is taking a flexible whalebone bougie, introducing it between the membrances and the uterine wall, high up into the uterus, and sweeping it gently around for one or two inches in either direction. This has not failed me in any instance in bringing on labor.

WILLIAM S. GARDNER, M. D.,
410 *Hanover St.* Secretary.

